# Thalamic volume differentiates multiple sclerosis from neuromyelitis optica spectrum disorder: MRI-based retrospective study

**DOI:** 10.3389/fneur.2024.1491193

**Published:** 2025-01-03

**Authors:** Manal Alosaimi, Hatham Alkanhal, Saleh Aldeligan, Nuha Alkhawajah, Alaa Albishi, Bander Hilabi, Salman Aljarallah

**Affiliations:** ^1^Department of Radiological Sciences, College of Applied Medical Sciences, King Saud University, Riyadh, Saudi Arabia; ^2^Department of Medicine, College of Medicine, King Saud University, Riyadh, Saudi Arabia; ^3^Department of Health Rehabilitation Sciences, College of Applied Medical Sciences, King Saud University, Riyadh, Saudi Arabia; ^4^Department of Medical Imaging Administration, King Saud Medical City, Riyadh, Saudi Arabia

**Keywords:** multiple sclerosis, neuromyelitis optica, thalamus, volume, CAT12, MRI

## Abstract

Multiple sclerosis (MS) and neuromyelitis optica spectrum disorders (NMOSD) are distinct demyelinating diseases of the central nervous system, each characterized by unique patterns of motor, sensory, and visual dysfunction. While MS typically affects the brain and spinal cord, NMOSD predominantly targets the optic nerves and spinal cord. This study aims to elucidate the morphometric differences between MS and NMOSD by focusing on gray matter volume changes in specific brain regions. We also examined if temporal changes in follow-up MRI differentiate the two disorders. We analyzed anatomical T1-weighted MRI scans from 24 patients with NMOSD and 25 patients with MS using the CAT12 toolbox. Our analysis revealed significant differences in gray matter structure between the two patient groups. Notably, the thalamus was found to be consistently smaller in patients with MS compared to those with NMOSD. This finding aligns with previous research highlighting thalamic atrophy as a hallmark of MS and further underscores the thalamus’s role in the disease’s pathology. These results provide valuable insights into the distinct neuroanatomical features of MS and NMOSD, contributing to a better understanding of the mechanisms underlying these diseases. The study also emphasizes the importance of advanced imaging techniques in differentiating between MS and NMOSD, which may have implications for diagnosis and treatment strategies.

## 1 Introduction

Immune-mediated diseases are among the most complicated and overlapping disorders in terms of pathophysiology, involvement of multiple organ systems, and clinical presentation. Of special interest is Multiple sclerosis (MS), the most common immune-mediated disease of the central nervous system (CNS). MS is a complex disorder characterized by CNS inflammation, demyelination, and axonal damage (1, 2). Patients with MS may exhibit symptoms due to inflammation in different parts of the central nervous system, but optic neuritis and myelitis are frequently observed as initial presentations. The contemporary diagnosis of MS typically requires the presence of clinical symptoms consistent with a relapse in addition to an MRI demonstrating typical MS plaques in characteristic locations in order to fulfill the widely accepted McDonald’s criteria (3). In some cases where there is doubt, ancillary neurophysiological tests or cerebrospinal analysis can be performed. Of note, the McDonald’s criteria are developed to diagnose MS in patients who have pictures highly suggestive of MS and not to differentiate MS from other diseases.

While the diagnosis of MS can be straightforward in many cases, it is not uncommon for MS to be incorrectly diagnosed in patients who have other mimicking disorders. The list of disorders that can present similarly to MS is long (4). Among those is neuromyelitis optica spectrum disorder (NMOSD), which shares with MS the relapsing–remitting course and the common involvement of the optic nerve and spinal cord as pathologic targets. NMOSD is another immune-mediated inflammatory disorder of the CNS that causes demyelination and axonal loss. However, there are major differences in immunopathogenesis as NMOSD is commonly caused by Aquaporin-4 (AQP4) antibodies in most patients, and the primary target is the astrocytes. These antibodies against AQP4 can be measured in the serum and are seen in the majority of patients with NMOSD (5). Some patients with NMOSD may not have AQP4 antibodies; instead, they might have Myelin oligodendrocyte glycoprotein (MOG) antibodies or no identifiable antibodies (double-negative). Patients with NMOSD, specifically those who are Anti-APQ4-positive, tend to have more severe attacks leading to significant visual loss and paralysis compared to patients with MS (6), highlighting the importance of early recognition and initiation of treatment.

Although MS and NMOSD overlap in many aspects, MRI can reveal some differences. In MS, brain lesions are often oval, seen in the cortical/juxtacortical and infratentorial regions and perpendicular to the lateral ventricles (Dawson’s fingers) (4). Spinal cord lesions are usually peripherally located and less than three vertebral segments in length. In NMOSD, lesions are usually peri-ependymal along the lateral ventricles, with pencil-like enhancement, and are longitudinally extensive in the spinal cord (7). However, some overlap in clinical features and imaging is still seen, making it sometimes difficult to differentiate these two entities based on routine MRI images. This is especially true in seronegative NMOSD cases. Misdiagnosing NMOSD as MS or vice versa could have serious consequences. For example, delaying the proper treatment of MS or NMOSD can lead to the accumulation of irreversible disability. Moreover, treating patients with NMOSD using MS treatment can lead to devastating consequences as MS treatments are known to exacerbate NMOSD. Newer MRI techniques showing cortical lesions, the central vein sign, and paramagnetic rim lesions could aid in differentiating the two conditions (8, 9).

It is thought that brain atrophy could be another possible differentiating imaging feature. Patterns of CNS atrophy differ between MS and NMOSD patients (10). Whether the longitudinal annualized brain atrophy rate is similar in MS and NMOSD is a matter of debate (10, 11). It is thought that, unlike MS, localized rather than global CNS degeneration is seen in NMOSD. However, regional gray matter atrophy has been reported in both (12, 13). Advanced imaging techniques, such as voxel-based morphometry, have been employed to quantify regional gray matter atrophy over the course of the disease (14, 15). For example, the computational anatomy toolbox (CAT12)^1^ for SPM (Statistical Parametric Mapping software)^2^ has been introduced, offering a fast and easy for brain segmentation.

In addition to its clinical importance as it correlates with disability, it might be of diagnostic significance (16, 17). Among different gray matter areas in the brain, of special interest is the thalamus, a major relay nucleus conveying different types of pathways to the cortex. It is thought that thalamus volume loss could be representative of net CNS damage and that it is a more sensitive measure than whole brain volume (10, 18).

To our knowledge, few studies have compared brain atrophy in MS versus NMOSD, and none in Saudi Arabia. In this paper, we compare whole brain, gray matter, and white matter volumes in MS versus NMOSD patients utilizing the CAT12 segmentation toolbox. We aimed to test whether it is possible to differentiate MS from NMOSD patients using different brain volume measures. In addition, we tested the hypothesis that the rate of atrophy is faster in MS compared to NMOSD by assessing the degree of volume change in a follow-up MRI.

## 2 Materials and methods

### 2.1 Participants

This retrospective study assessed patients who visited the neurology outpatient clinic at King Saud University Medical City. The medical city is a tertiary hospital that hosts a specialized clinic in MS and neuroimmunological disorders. Patients with NMOSD and MS who were on immunomodulatory treatments were included. The subject is included in the MS group if they met the diagnostic criteria for McDonald 2017 criteria for relapsing MS and in the NMOSD group if the International Panel for NMOSDDiagnosis (IPND) criteria were met regardless of antibody status. Patients with MOG antibodies were included in the NMOSD group if they fulfilled the seronegative NMOSD criteria. Antibodies against AQP-4 and MOG have to be present using cell-based assays (CBAs) as they have been shown to offer higher sensitivity and specificity (19, 20).

To be included in the study, subjects must have undergone at least two MRI scans. Both sessions (baseline and follow-up) must include T1-weighted images with fast spin-echo gradient-echo (FSEGE) sequences. The follow-up MRI for each subject has to be performed 6 months or after in order to capture slow volume loss. The subjects were excluded if any of the following criteria were met: incomplete clinical information, presence of another neurological condition that is known to affect brain structure, lack of follow-up MRI session, lack of the FSEGE MRI sequences at baseline or follow-up MRI, corrupt data following DICOM to NIfTI file conversion or reading, and errors upon loading the NIfTI files. The study obtained ethical approval from the King Saud University Ethics Committee.

### 2.2 Data collection

Three-dimensional T1-weighted images were collected from the Picture Archiving and Communication System (PACS) at the King Saud University Medical City.

### 2.3 Data processing

The images were processed with the software package the Computational Anatomy Toolbox (CAT12, see text footnote 1) for SPM12 (Statistical Parametric Mapping software, see text footnote 2) using MATLAB version (R2020a). CAT12 delivers an accurate and robust and can be considered a fast and reliable alternative to other approaches for neuroimaging analysis. Initially, all images were checked for the same orientation (anterior commissure–posterior commissure axis) as the priors of SPM. The longitudinal longitudinal processing stream for VBM was used, and the MS and NMOSDgroups were processed separately. The structural images were segmented following the standard procedure (21), using CAT12 implemented in SPM12 to generate gray matter, white matter, and cerebrospinal fluid images. The individual GM volume was segmented into subcortical structures based on the CAT12 Neuromorphometrics atlas (Neuromorphometrics Inc., Somerville, MA, USA). Two experts in mapping analysis software checked the output segmentation files, and subjects with poor segmentation quality were excluded from the study. Finally, the XML files for the two groups were created and saved as CSV files for statistical analysis.

### 2.4 Statistical analysis

**Figure 1:**
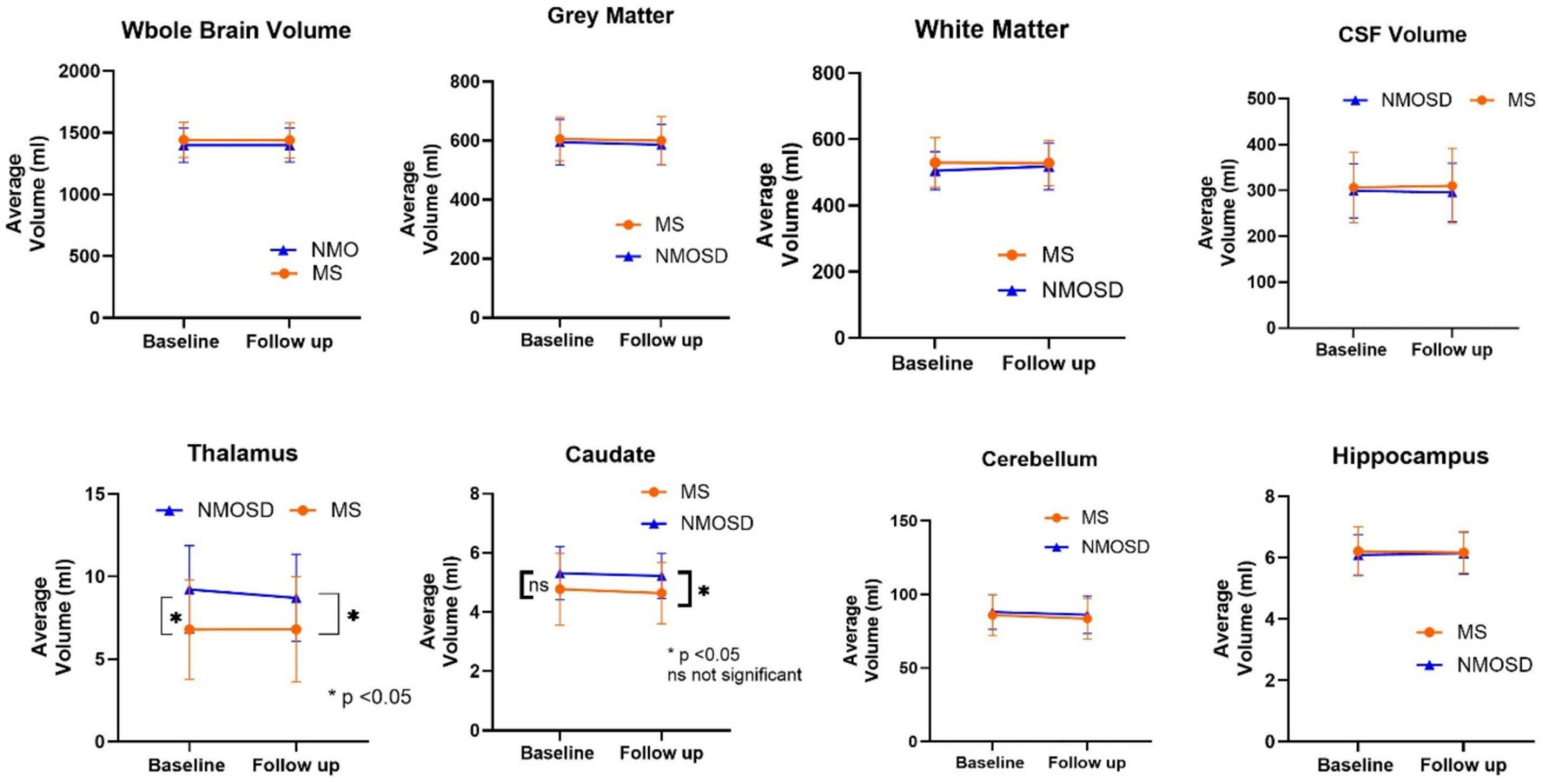
Comparison of mean whole and regional brain volumes between MS and NMOSD patients at baseline and follow-up.

Data were analyzed using Minitab Statistical Software Version 21.4.2.0. For categorical data, Chi-square and Fisher’s Exact Tests were used as appropriate. Continuous data for each variable from each patient for baseline and follow-up scans were performed. Quantitative data were analyzed for each variable from each patient for baseline and follow-up scans. To accept or reject the null hypothesis, we performed the following tests according to the measured outcome. Student’s *t*-test was used to compare the means of the regional brain volumes at baselines and at follow-up. Also, the student *T*-test was utilized to compare brain volumes between NMOSD, and MS. A one-way ANOVA was the test of choice for the comparison between the means of brain volume across patients with NMOSD with different antibody statuses. A *p*-value of < 0.05 was considered statistically significant and sufficient to reject the null hypothesis. A binary logistic regression analysis was performed to determine the best combination of parameters to differentiate between scans for the groups and predict the disease classification based on different parameters (Figure 1).

## 3 Results

### 3.1 Demographic and clinical characteristics

Patients with MS sclerosis were younger than patients with NMOSD. While most patients with NMOSD were females (71%), the MS group constituted an equal number of males and females. The mean disease duration was longer in the MS cohort compared to NMOSD, although this was not statistically significant. Demographic and clinical characteristics are shown in Table 1. Patients with NMOSD were examined for the presence of antibodies. About 54% of patients tested positive for aquaporin-4 (AQP4) antibodies, and 25% tested positive for MOG antibodies. The remaining did not have either of these antibodies and were considered double negative (DN). The presence and distribution of structural abnormalities (demyelinating lesions) in the baseline MRI were compared between the MS and NMOSD cohorts, as shown in Table 2. In terms of clinical features, all patients with MS had optic neuritis compared to 75% of Patients with NMOSD. More patients with MS had a history of transverse myelitis compared to patients with NMOSD (64% vs. 41%), although this was not statistically significant (*p* = 0.156). Also, more patients with MS had a history of symptoms localizable to the cerebrum or brainstem compared to patients with NMOSD. In terms of treatment, all patients were treated with variable forms of immunomodulation. About 75% of patients with NMOSD were treated with rituximab or mycophenolate. This contrasts with patients with MS, in whom 28% were on rituximab and none were on mycophenolate.

**Table 1:** Demographics and clinical characteristics.

Variable	MS (*N* = 25)	NMOSD (*N* = 24)	*p*-value
Age (mean ± SD)	27.12 ± 6.3	35.43 ± 13.1	0.01*
Female (%)	12 (48.0%)	17 (70.8%)	0.15 **
Disease duration (mean ± SD)	6.48 ± 3.54	4.96 ± 3.42	0.13*
Antibodies			
Aquaporin-4 (AQP4)	n/a	13 (54.2%)	n/a
MOG antibodies		6 (25.0%)	
Double negative (DN)		5 (20.8%)	
Clinical features			
Optic neuritis	25 (100%)	18 (75%)	0.01 **
Transverse myelitis	16 (64%)	10 (41%)	0.16 **
Area postrema	0% (0)	2 (8.33%)	0.24 **
Cerebral attacks	16 (64%)	2 (8.33%)	< 0.05 **
Brainstem attacks	19 (67%)	5 (20.83%)	< 0.05 **
Diencephalic attacks	4 (16%)	1 (4.17%)	0.35 **
Current DMT			
Rituximab	7 (28%)	13 (54.17%)	0.001 **
Mycophenolate	None	5 (20.83%)	
Other	18 (72.00%)	6 (25.00%)	
Treatment-naïve	5 (20.00%)	14 (58.33%)	0.009 **
Months between MRIs (mean ± SD)	14.24 (±2.78%)	21.07 (±10.33)	0.006
	*n* = 21	*n* = 20	
Follow up MRI			
6–12 months	4 (16%)	3 (12.5%)	
12–18 months	16 (64%)	6 (25%)	
18–24 months	1 (4%)	6 (25%)	
> 24 months	4 (16%)	9 (37.5%)	

* Student *t*-test, ** Fisher’s exact.

**Table 2:** Lesion distribution in baseline magnetic resonance imaging.

Variable	MS (*N* = 25)	NMOSD (*N* = 24)	*p*-value (Fisher’s exact)
Periventricular lesion	23 (92.00%)	7 (29.17%)	< 0.05
Cortical lesions	21 (84.00%)	8 (33.33%)	< 0.05
Cerebellar lesions	20 (80%)	3 (12.5%)	< 0.05
Brainstem lesions	21 (84%)	4 (16.67%)	< 0.05
Optic nerve lesion	22 (88%)	15 (62.50%)	0.05
Cervical cord lesion	20 (80%)	12 (50.00%)	0.04
Thoracic cord lesion	20 (80%)	8 (33.33%)	0.001
Conus medullaris lesion	3 (12.00%)	3 (12.50%)	1.0
Gadolinium enhancement	3 (12.00%)	4 (16.67%)	0.7

### 3.2 White matter lesions

We also examined the presence of structural abnormalities on MRI between the two groups. As expected, patients with MS had more areas affected through the central nervous system, but only 12% of patients had enhancing lesions or lesions in the conus medullaris, which is similar to the NMOSD groups. The common areas affected by NMOSD are optic nerves (62.5%) and cervical spinal cord (50%). Only three patients in the NMOSDgroup (12.5%) have cerebellar lesions compared to 80%.

### 3.3 Whole and regional brain volume

The quantitative values of variable regional volume NMOSD and MS groups in baseline and follow-up scans are shown in Table 3. We examined the volumes at baseline and follow-up MRIs. Looking at baseline MRIs, there was no statistically significant difference in the whole brain volume, white matter, gray matter, or CSF volumes that can be observed between patients with MS or NMOSD, although these parameters tend to be higher among patients with MS. However, the cerebral white matter (excluding infratentorial white matter) was significantly larger in MS patients compared to those with NMOSD (69.3 ± 10.3 vs. 62.8 ± 9.3, *p* = 0.02). This relation did not change when we assessed the volume in the follow-up MRI. Nevertheless, the most striking difference between MS and NMOSD was in the thalamic volumes, where MS patients had a smaller thalamic size compared to NMOSD in baseline (average of 6.8 ± 3.0 in MS compared to 9.2 ± 2.6 in NMOSD with a *p*-value of 0.004. When we examined NMOSD subtypes, we observed that patients with seronegative NMOSD (*n* = 5) had the largest thalamic volume (average 10.96 mL ±2.18) followed by MOG-positive NMOSD (Average 9.1 mL ± 2.3 mL) and then AQP4-positive patients with an average thalamic volume of 8.6 ± 2.8 mL). (Table 4). This difference between average thalamic volume between MS and NMOSD groups persisted in follow-up MRI (6.8 ± 3.2 and 8.7 ± 2.6, *p* = 0.03). In order to understand the impact of other variables, we ran a multivariate regression logistic regression analysis examining the relation between variable parameters and the diagnosis of MS vs. NMOSD (Table 5). The unadjusted model confirmed the statistically significant difference in the baseline scan between the two groups (OR 1.35, *p* = 0.009). This relationship was not changed by adjusting for age and disease duration (OR 1.35, *p* = 0.03). In addition, the ROC curve analysis showed that the thalamic volume resulted in 70.6% sensitivity and 64.3% specificity and an area under the curve of 0.73 in differentiating NMOSD and MS groups in the baseline scan (Table 6). Therefore, we estimated a thalamic volume smaller than 7.06 mL to favor the diagnosis of MS rather than NMOSD. Also, cerebral white matter volume was larger in MS compared to NMOSD (OR 0.9, p = 0.03). However, this relationship was not statistically significant after the introduction of age in the model (OR 0.97, *p* = 0.4). The caudate volume was smaller in MS patients compared to NMOSD. However, this was only seen in follow-up scans. A smaller caudate volume in the follow-up scan was associated with an increase in the odds of the diagnosis of MS (OR 2.5, *p* = 0.04, 95% CI between 1.03 and 6.25). Cerebellar and hippocampal volumes were not different between MS and NMOSD (Table 7).

**Table 3:** Quantitative values of regional brain volumes comparing MS to NMOSD at baseline and follow up.

Average regional volume (mean ± SD)	Baseline MRI			Follow-up MRI		
	MS (*N* = 25)	NMOSD (*N* = 24)	*p*	MS (*N* = 25)	NMOSD (*N* = 24)	*p*
Whole brain volume	1441.7 ± 142.8	1399.3 ± 138.2	0.29	1438.8 ± 142.7	1399.9 ± 137.1	0.34
Gray matter	605.6 ± 73.8	595.2 ± 76.9	0.63	600.2 ± 81.1	595.2 ± 76.9	0.52
White matter	529.8 ± 75.6	504.8 ± 57.3	0.20	528.3 ± 68.3	518.1 ± 70.2	0.61
CSF	306.3 ± 76.6	299.2 ± 58.9	0.72	310.3 ± 81.6	295.6 ± 63.8	0.49
Caudate	4.8 ± 1.2	5.3 ± 0.9	0.08	4.6 ± 1.0	5.2 ± 0.8	0.03
Cerebellum	85.9 ± 13.9	88.1 ± 11.8	0.56	83.6 ± 13.9	86.2 ± 12.8	0.49
Cerebral white matter	69.3 ± 10.3	62.8 ± 9.3	0.02	69.2 ± 9.3	63.1 ± 8.6	0.02
Hippocampus	6.2 ± 0.8	6.1 ± 0.7	0.58	6.2 ± 0.7	6.1 ± 0.7	0.92
Thalamus	6.8 ± 3.0	9.2 ± 2.6	0.004	6.8 ± 3.2	8.7 ± 2.6	0.03

**Table 4:** Regional volumes comparing NMOSD subtypes at baseline MRI.

Regional volumes (mean ± SD)	MS (*N* = 25)	NMOSD (*N* = 24)	AQP-NMOSD (*N* = 13)	MOG-NMOSD (*N* = 6)	DN-NMOSD (*N* = 5)	*p* value * (MS vs NMOSD)	*p* value *(MS vs AQP4)	*p* value *(MS vs MOG)	*p* value *(MS vs DN)
Whole brain volume	1441.7 ± 142.8	1399.3 ± 138.2	1372.5 ± 153.8	1389.6 ± 122.1	1480.3 ± 97.7	0.29	0.17	0.42	0.57
Gray matter	605.6 ± 73.8	595.2 ± 76.9	569.61 ± 65.6	608.9 ± 93.7	645.4 ± 67.2	0.63	0.14	0.93	0.28
White matter	529.8 ± 75.6	504.8 ± 57.3	482.7 ± 64.4	514.2 ± 32.9	551.1 ± 25.1	0.20	0.06	0.63	0.54
CSF	306.3 ± 76.6	299.2 ± 58.9	320.2 ± 67.06	266.5 ± 37.2	283.8 ± 36.8	0.72	0.58	0.23	0.53
Caudate	4.8 ± 1.2	5.3 ± 0.9	5.19 ± 0.9	5.08 ± 1.0	5.9 ± 0.4	0.08	0.27	0.58	0.05
Cerebellum	85.9 ± 13.9	88.1 ± 11.8	82.6 ± 10.7	92.3 ± 11.9	97.3 ± 6.9	0.56	0.47	0.31	0.09
Cerebral white matter	69.3 ± 10.3	62.8 ± 9.3	59.9 ± 7.6	67.3 ± 12.2	65.0 ± 8.5	0.02	0.01	0.68	0.39
Hippocampus	6.2 ± 0.8	6.1 ± 0.7	5.9 ± 0.6	6.0 ± 0.6	6.7 ± 0.5	0.58	0.24	0.56	0.21
Thalamus	6.8 ± 3.0	9.2 ± 2.6	8.6 ± 2.8	9.1 ± 2.3	10.9 ± 2.2	0.004	0.08	0.095	0.07

**Table 5:** Logistic regression and ROC curve result for structural volume in baseline MRI.

Parameter	Unadjusted			Adjusted for age, gender		
	OR	95% CI	*p* value	OR	95% CI	*p* value
Whole brain volume	1.00	0.99–1.00	0.29	1.00	0.99–1.00	0.48
Gray matter	1.00	0.99–1.01	0.624	1.00	0.99–1.01	0.50
White matter	0.99	0.99–1.00	0.200	0.99	0.98–1.01	0.35
CSF	1.00	0.99–1.01	0.71	0.99	0.98–1.00	0.27
Caudate	1.65	0.92–2.94	0.09	1.75	0.91–3.36	0.09
Cerebellum	1.01	0.97–1.06	0.55	1.04	0.98–1.11	0.16
Cerebral white matter	0.93	0.87–0.99	0.03	0.96	0.89–1.05	0.40
Hippocampus	0.80	0.36–1.75	0.57	0.95	0.36–2.46	0.91
Thalamus	1.35	1.08–1.70	0.009	1.36	1.06–1.74	0.01

**Table 6:** Logistic regression and ROC curve results for baseline thalamus.

Scan	Parameter	OR	95% CI	*p* value of (LGA)	Sensitivity %	Specificity %	AUC
Baseline	Thalamus	1.35	1.07–1.69	0.009	70.60%	64.30%	0.73

**Table 7:** Mean change in brain volumes in follow up MRI comparing MS to NMOSD.

	MS (*N* = 25) (mean ± SD)	NMOSD (*N* = 24) (mean ± SD)	p*
Average change in whole brain volume			
Absolute (ml)	−2.96 ± 10.5	0.73 ± 9.6	0.21
Relative (%)	−0.20 ± 0.7	0.6 ± 0.7	
Average change in gray matter volume			
Absolute (ml)	−5.4 ± 32.1	−8.89 ± 35.1	0.72
Relative (%)	−0.91 ± 5.4	1.2 ± 5.6	
Average change in white matter volume			
Absolute (ml)	−1.5 ± 29.9	13.27 ± 46.1	0.19
Relative (%)	−0.05 ± 5.9	2.7 ± 9.2	
Average change in CSF volume			
Absolute (ml)	3.95 ± 22.8	−3.65 ± 22.9	0.25
Relative (%)	1.2 + 8.3	−1.3 ± 7.7	
Average change in caudate volume			
Absolute (ml)	−0.13 ± 0.6	−0.09 ± 0.5	0.84
Relative (%)	−0.27 ± 16.7	−0.98 ± 9.7	
Average change in cerebellum volume			
Absolute (ml)	−2.3 ± 6.0	−1.9 ± 7.9	0.83
Relative (%)	−2.4 ± 7.5	−2.0 ± 9.19	
Average change in cerebral white matter volume			
Absolute (ml)	−0.14 ± 3.4	0.28 ± 2.4	0.62
Relative (%)	−0.09 ± 4.8	0.6 ± 3.6	
Average change in hippocampus volume			
Absolute (ml)	−0.04 ± 0.3	0.05 ± 0.4	0.38
Relative (%)	−0.3 ± 4.7	1.1 ± 7.7	
Average change in thalamus volume			
Absolute (ml)	0.01 ± 2.7	−0.5 ± 2.4	0.48
Relative (%)	9.2 ± 53.2	−0.91 ± 31	

* student *t*-test.

### 3.4 Follow-up MRI

In order to examine the hypothesis that patients with MS lose brain volume faster than NMO, we compared brain MRIs done 6 months or more from the baseline MRI. At follow-up, on average, both patients with MS and NMOSD suffered some reduction in the gray matter volume, caudate volume, and cerebellar volume. However, the differences were not statistically significant. Patients with MS have lower whole brain volume, cerebral white matter volume, and hippocampal at follow-up, while NMOSD patients had lower CSF volume and thalamic volumes at follow-up compared to baseline. Important to note that these differences are small and not statistically significant.

## 4 Discussion

In this observational study, we retrospectively used MRIs performed for clinical purposes to identify differences in the structural volume between patients with MS and NMOSD. We did not exclude patients with NMOSD who tested negative for NMOSD and MOG antibodies. This study identified that, indeed, significant differences do exist between MS and NMOSD, even if clinical presentations are more or less similar. While some of these differences, including gross abnormalities such as plaques, can be easily visually seen in routine MRI, we identify more subtle differences in the deep gray matter volume that help differentiate MS from NMOSD, which helps in the management. The most consistent finding in this study was that a smaller thalamic volume in patients with MS was a strong predictor for the diagnosis of multiple sclerosis and is a good marker to differentiate MS from NMOSD.

Brain imaging, specifically MRI, is a cornerstone in the diagnosis and management of neuroinflammatory disorders such as MS and NMOSD. In addition to the traditional inflammatory plaques that are observed in patients with MS, loss of brain volume has been recognized in imaging for more than two decades (22). Earlier research identified that cerebral atrophy occurs faster in patients with MS than in healthy people (23). The deep gray matter, specifically the thalamus, has been a focus of extensive research as it has been consistently shown to be smaller in patients with multiple sclerosis (18). Even studies looking at children with MS found smaller thalamic volume compared to patients with MOG-associated disorder (24). Importantly, thalamic volume loss in MS has been associated with disability and cognitive impairment (25, 26). The etiology of thalamic volume loss in MS is a subject of debate. Demyelinating lesions in the thalamus are not uncommon and can be seen in the majority of patients, especially when ultra-field MRI is used (27). It would have been plausible that a disease like MS that causes widespread demyelinating lesions or plaques in the thalamus is a significant driver of volume loss. However, studies examining this have led to different conclusions. In a study reported by Mehndiratta et al., thalamic volume loss does not appear to be directly related to thalamic demyelinating lesions when looked at using an ultra-high field (7-Tesla) MRI. In this study, the thalamic volume did not differ between patients with and without thalamic lesions (28). A postmortem MRI study confirmed that demyelinating thalamic lesions do not contribute significantly to thalamic volume loss (29). However, it has been suggested that only certain types of thalamic demyelinating lesions contribute to thalamic volume loss, while others do not. For example, in a study utilizing 7 T tesla MRI, the overall thalamic volume negatively correlated with the number and the volume of periventricular thalamic demyelinating lesions, but not ovoid thalamic lesions (27). Nevertheless, the lack of a strong association between thalamic volume and thalamic plaques has led researchers to investigate the potential role of other factors within the thalamus, such as neuronal damage, axonal loss, and progressive neurodegenerative mechanisms (18, 30, 31). Histologic studies performed on patients with MS revealed that thalamic neuronal density is reduced by about 22–33% compared to healthy controls (32). The authors suggested that the loss of certain types of neurons, such as interneurons, could contribute to the volume loss (29). Mechanisms associated with degeneration in multiple sclerosis include iron accumulation, excess calcium, glutamate excitotoxicity, mitochondrial dysfunction and oxidative stress (33). This explains the lack of impact of disease-modifying therapies on the thalamic size as disease-modifying treatments (DMTs) target inflammatory patholgoy with no meaningful impact on neurodegeneration. In addition to processes within the thalamus, thalamic atrophy is likely aggravated by injury occurring outside, either inflammatory or degenerative. This distinction is significant because, unlike NMOSD or MOG, which tend to be focal, MS causes widespread inflammation and neurodegeneration throughout the central nervous system, affecting multiple neuroanatomical pathways. Therefore, the thalamus, as a major hub for axons transmitting to and from other CNS structures, appears to be particularly susceptible to degeneration secondary to an injury outside the thalamus. Interruption of thalamic afferents or efferents can lead to degeneration, which may affect axons traveling away from the thalamus (“retrograde”) or toward the thalamus (“anterograde”) (34). In addition, a process of transsynaptic degeneration has been observed in MS. For example, in patients with MS, thalamic volume loss can be seen in patients who suffered an episode of optic neuritis (35). In our study, compared to patients with NMOSD, those with MS exhibited more periventricular and cortical lesions, as well as increased involvement of the brainstem and spinal cord. Therefore, the thalamic volume changes in this cohort could be a manifestation of extra-thalamic injury. Therefore, the larger thalamic volume in NMOSD compared to MS was not an unexpected finding given the smaller number of lesions outside the thalamus. This is also in line with previous studies in NMOSD. A prior study of patients with APQ4-positive NMOSD found that the deep gray matter structures are not different from healthy controls (36). However, a more recent study of 85 patients with NMOSD reported that thalamic volumes are smaller compared to healthy controls in NMOSD patients (37). A similar finding was reported in addition to atrophy of the white matter and caudate in a larger study from Korea, which looked at patients with NMOSD who had cognitive impairment but not in patients without cognitive impairment (38). Other studies have reported altered volumes of thalamic nuclei in patients with NMOSD who had optic neuritis (39) or pain intensity (40). In general, our study is in agreement with the literature that thalamic volume is more pronounced in MS regardless of lesion burden or clinical attack, while in NMO, thalamic atrophy is less pronounced and is related to clinical attacks rather than diffusive pathology. Studies on patients with MOG antibody disorder (MOGAD) have reported variable changes affecting the deep structures. A study comparing 20 patients with MOGAD to patients with NMOSD and MS found that patients with MOGAD had a lower volume of deeper structures, which was hypothesized to be related to persistent WM lesions (41). A study of 17 patients with MOGAD reported atrophy in the thalamus compared to healthy controls in addition to atrophy affecting frontal and temporal lobes, insula, and hippocampus (42). A similar finding was reported in another study looking at a subgroup of patients with relapsing MOGAD (43). Of note, our study included patients with MOG antibodies only if they fulfill the IPND criteria for NMOSD, and it does not necessarily translate to recent studies that defined MOGAD using the 2023 international criteria (44).

The population in this cohort reasonably represents contemporary patients with the studied disorders. The average age of patients in these groups is consistent with the population of NMOSD and MS in Saudi Arabia (45–47). There is an overrepresentation of females in the NMOSD group, which is a well-known phenomenon in NMOSD. In some NMOSD cohorts, females constitute almost 90% of the patients (45). The proportion of females in the MS cohort is lower than in the typical MS cohort. This is probably related to the smaller sample size and the fact that many patients were excluded due to a lack of appropriate MRI sequences for this study. These differences in age and gender between the groups did not impact the volume of the thalamus, as shown in the multivariate analysis. In terms of clinical presentation, optic neuritis and transverse myelitis were the most common manifestations in both groups, reflecting the disease’s predilection for these areas and aligning with its natural history. However, in this study, all patients with MS have experienced an episode of optic neuritis, and almost 90% of them had optic nerve abnormality on MRI, which is higher than expected in a typical MS cohort. We hypothesize that this is likely due to the majority of referrals to our hospital coming from active, large tertiary neuro-ophthalmology practices. This is an important factor, as previous studies in NMO-associated changes in the lateral geniculate nucleus of the thalamus have assumed that these changes are secondary to anterograde degeneration in patients with optic neuritis in NMOSD (39, 48). To determine if the higher prevalence of optic neuritis in the MS group influenced the outcomes, we performed a sensitivity analysis looking only at patients with optic neuritis in both groups. The thalamic volume remained a good predictor for the diagnosis of NMOSD with an odds ratio (OR) of 1.4 (*p*-value of 0.014 and 95% CI between 1.1 and 1.8). Thus, optic neuritis does not appear to account for the observed difference. The lack of patients with area postrema syndrome in MS and the presence of 2 patients with NMOSD is consistent with the literature (49). More patients with MS had attacks affecting the brainstem (67%) compared to patients with NMOSD (21%). Also, more patients with MS had MRI lesions in the brainstem compared to NMOSD (84% vs. 16.7%, respectively). This is close to what has been previously reported. For example, a multicenter study of 258 patients with NMOSD reported that 81 patients (31.4%) had symptoms localizable to the brain stem (50). A 2016 study of 50 patients with MOG-IgG-positive transverse myelitis or optic neuritis reported that 15 patients (30%) had a history of brainstem encephalitis (51). The thalamus volume remained smaller in patients with MS (average 6.9 mL) compared to NMOSD (average 9.2 mL) if only patients without brainstem attacks or brainstem lesions were included. Therefore, in this study, the higher number of patients with MS who had brainstem lesions or brainstem attacks did not impact that difference in the thalamic volume. We also examined the impact of immunomodulatory treatment in this cohort. As the vast majority of NMOSD patients in our recentre are on treatment, we excluded patients with MS who are not treated to avoid any confounding effects. While both diseases are immune-mediated and are generally treated with immunosuppressants, specific strategies in immunosuppression differ as some MS treatments exacerbate NMO. We examined that being on treatment might ameliorate the brain volume differences between the disorders. However, the OR did not change and remained statistically significant after adjusting for the current treatment in the multivariable model. The average thalamic volume of patients with MS remained significantly smaller when compared to only patients on rituximab in both groups. It is important to note that patients with NMOSD in our cohort are more likely to start rituximab as a first line (58% vs. 20%). Traditionally, patients with multiple sclerosis, including some of the patients in this cohort, are started on a lower-efficacy DMT and then are changed to a higher-efficacy treatment if the disease progresses. In this study, the duration of the current DMT was not examined. However, we did examine the disease duration, which is the time since the first clinical relapse in both groups, and we did not find a significant difference in both groups. It was relatively short in both groups (an average of 6.5 years in MS vs. 5 years in NMO). Therefore, it is likely that patients with MS who were escalated to rituximab were switched to it early on their disease course. Nevertheless, the impact of immunomodulation on the progression of the deep gray matter volume should examined in a prospective study. In this study, we selected MRI regardless of the most recent disease activity. Hence, the prevalence of gadolinium-enhancing lesions in both groups was low.

We tried to establish if the rate of global and regional brain volume loss would differentiate MS from NMO. In both groups, we looked at follow-up MRIs performed more than 6 months. We looked at absolute and relative differences between the initial and follow-up MRI but found no significant difference between MS and NMOSD groups. Other studies have shown that patients with MS tend to experience a faster loss of brain volume compared to healthy. In our study, whole brain volume was smaller by about 0.2% in follow-up MRIs done on average after 14 months. This is similar to the average volume loss seen in healthy controls and is lower than expected in patients with multiple sclerosis, as reported in previous studies where the average rate was 0.7–0.8% (18, 23). However, we believe this is likely due to the effect of treatment as this is similar to 0.19% loss of brain volume in patients with MS taking rituxmiab (52) or ocrelizumab, a medication with an efficacy similar profile to rituximab (53). Also, we only looked at one follow-up MRI rather than serial MRIs over a longer period, which might have shown more progressive atrophy in patients with MS.

This study has a few limitations. First, the sample size is not very large given that NMOSD is not a common disease in our population (47) although the size is comparable to other single-center studies (23, 46). The retrospective design resulted in variable times for the follow-up MRI within and between groups. Also, we had to exclude patients who had MRIs done using different protocols. Nevertheless, this makes the cohort in our study more representative of real-life situations and allows better generalizability of the results. Also, in this study, we did not include patients with MOG antibodies who they did not fulfill the criteria of NMOSD. As the study design utilized multiple comparisons, there is a risk of Type I error. However, this risk is low due to the relatively small number of comparisons, very few statistically significant results, strong biological plausibility, and supportive literature to the positive findings. Also, correction methods tend to be conservative and unnecessarily increase the risk of Type II error, which is more likely in such a small study.

## 5 Conclusion

This study shows that thalamic volume is smaller in patients with MS and can be used as a marker to differentiate MS from NMOSD in a real-world setting. A larger study is needed to confirm this finding.

## Data Availability

The datasets presented in this article are not readily available because of ethical and privacy restrictions. Requests to access the datasets should be directed to Prof. Ahmad A. Abdulmomen, aturk@ksu.edu.sa.

## Footnotes

^1^ http://www.neuro.uni-jena.de/cat/

^2^ http://www.fil.ion.ucl.ac.uk/spm/
